# Elevated PSA level as a warning of mesh rejection risk after combined laparoscopic totally extraperitoneal hernia repair and transurethral resection of prostate: Case report

**DOI:** 10.1016/j.ijscr.2019.04.037

**Published:** 2019-05-16

**Authors:** Adeodatus Yuda Handaya, Aditya Rifqi Fauzi, Victor Agastya Pramudya Werdana

**Affiliations:** a Digestive Surgery Division, Department of Surgery, Faculty of Medicine, Public Health and Nursing, Universitas Gadjah Mada/Dr. Sardjito Hospital, Yogyakarta 55281, Indonesia; b Faculty of Medicine, Public Health and Nursing, Universitas Gadjah Mada/Dr. Sardjito Hospital, Yogyakarta 55281, Indonesia

**Keywords:** Laparoscopy, Bilateral inguinal hernia, Prostate hypertrophy, Rejected mesh, Elevated PSA levels

## Abstract

**Background:**

Prostate hypertrophy and inguinal hernia are common health issues in men aged more than 50 years. Recently, many studies showed that prostatectomy and laparoscopic inguinal hernia repair could be performed at the same time. This report depicts mesh rejection after concomitant repair of bilateral direct inguinal hernia with TEP and TURP.

**Presentation of case:**

A 66-year-old man presented with bilateral direct inguinal hernia and enlargement of the prostate. Prostate biopsy shows atypical adenomatous hyperplasia accompanied by chronic inflammatory cells. Surgery was performed with laparoscopic TEP by using mesh and TURP. The patient underwent a combination of laparoscopic TEP and TURP. A single preoperative and postoperative dose of levofloxacin was administered. The laparoscopic TEP and TURP procedures were completed with no difficulty. Hematoma at the right inguinal occurred three weeks after the procedure. Bilateral inguinal abscess and sepsis occurred at 3-month follow-up. We performed laparotomy mesh evacuation and debridement, and found no recurrence of the hernia.

**Discussion:**

Combined laparoscopic TEP hernia repair and TURP are feasible procedures. Bladder erosion caused by mesh might be the cause of mesh infection. However, the elevated PSA levels in this patient can be a warning sign of chronic infection in the prostate which can lead to complications of surgery.

**Conclusions:**

Elevated PSA levels can be a warning before doing a combination of TEP and TURP surgery because they can show signs of infection. However, further research is needed to ascertain the relationship between elevated PSA levels and complications of combination TEP and TURP surgery.

## Highlights

• Mesh rejection after combined laparoscopic TEP and TURP.
• High PSA level before procedure.
• An abscess developed several months after operation.
• Might be caused by mesh erosion to viscera.
• Mesh evacuation and debridement had been performed.
• No recurrence of hernia was found after mesh evacuation.

## 1 Background

Inguinal hernias are one of the most common abnormalities for surgeons from generation to generation throughout the world. Over nearly a century, there have been many studies that describe the material used to close the hernia defect. Many hernia repair techniques are done but they have a high recurrence. Lately, increasingly popular laparoscopic surgical techniques have been used to treat inguinal hernias. These techniques reduce postoperative pain with faster recovery time and low recurrence rates. Two types of laparoscopic techniques that are often used are transabdominal preperitoneal (TAPP) and totally extraperitoneal (TEP) [1].

In recent years, the incidence of patients with prostate cancer has increased, and inguinal hernias are also common in prostate cancer patients. In addition, currently inguinal hernia is considered as one of the long-term complications of radical prostatectomy [2]. TEP has recurrence and morbidity rates that are equivalent to open and laparoscopic surgical techniques. TEP itself has advantages in diagnosing and repairing contralateral hernias that are not unexpected. The duration of operation and conversion rates tend to be low in proportion to operator experience [3].

We present a case report where the combination of laparoscopy and TURP was complicated by erosion of the mesh into viscera which was presented as an inguinal abscess. This case is reported by an operator with experience as a digestive surgeon. This research work has been reported in line with the SCARE checklist [12].

## 2 Case presentation

**Fig. 1:**
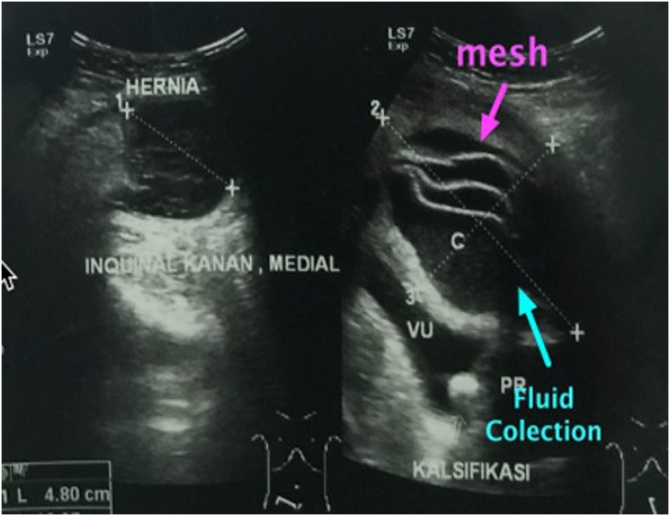
USG after 3-week post operation.

**Fig. 2:**
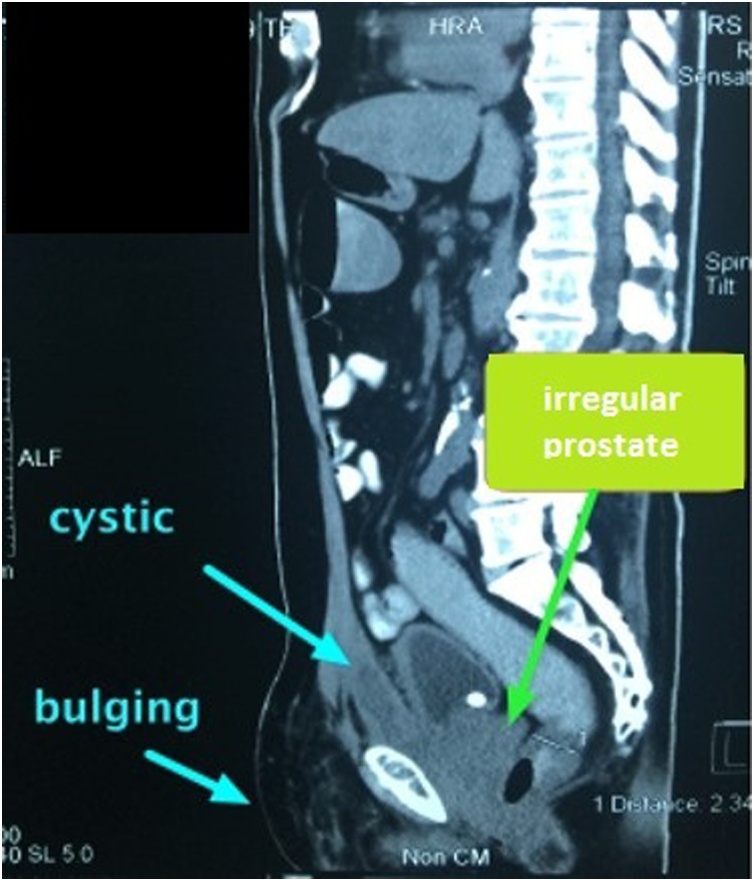
3-month follow-up CT Scan axial view showed mass on the prostate.

**Fig. 3:**
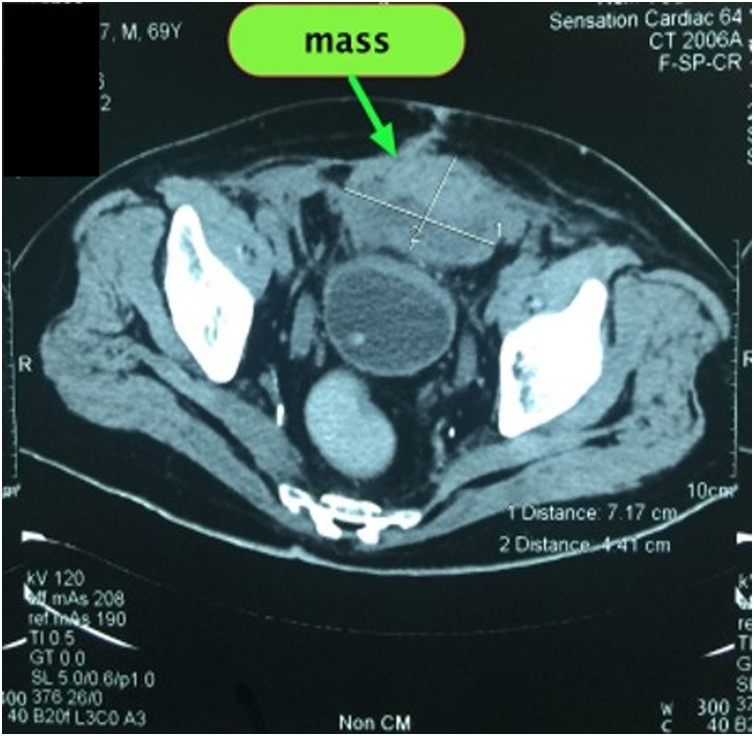
3-month follow-up CT Scan sagittal view showed mass in the abdomen.

**Fig. 4:**
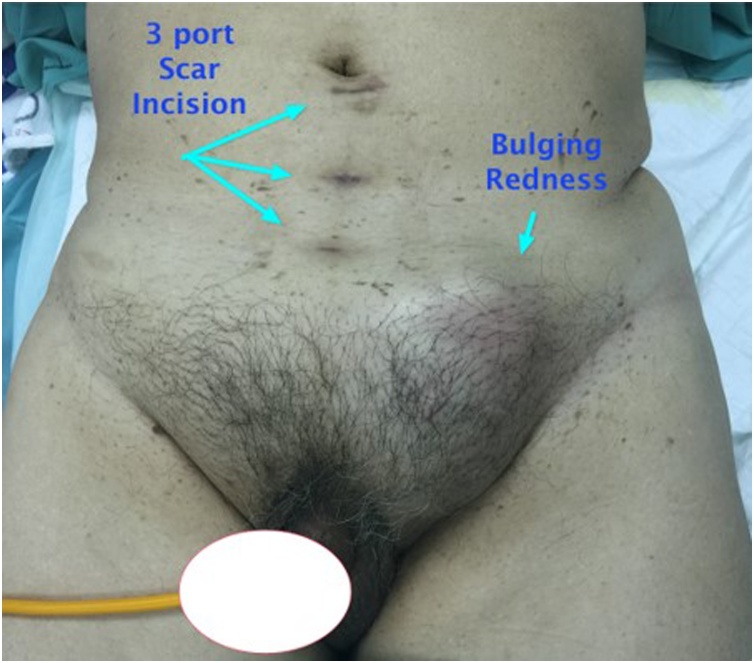
Port Scar and Inguinal Abscess.

**Fig. 5:**
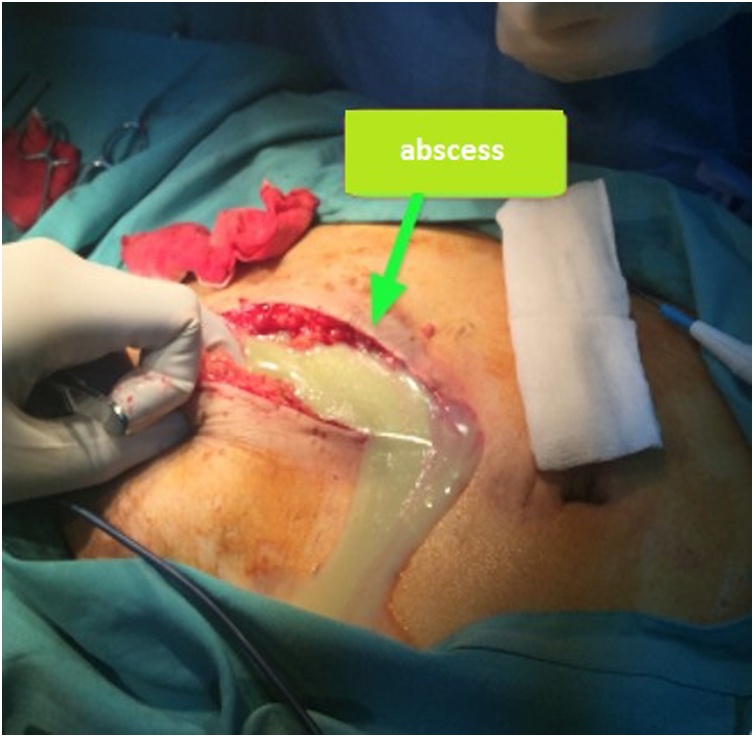
Intraoperative view during laparotomy showing abscess drainage.

**Fig. 6:**
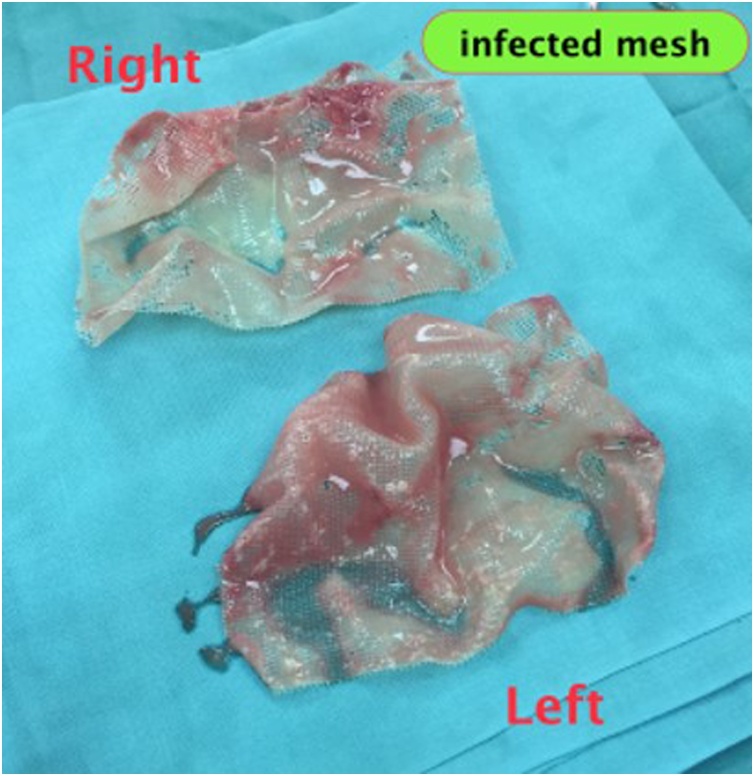
Infected mesh after evacuation.

A 66-year-old man presented with bilateral direct inguinal hernia and benign prostatic hyperplasia. Patient received a TEP (totally extraperitoneal) procedure using mesh and TURP (transurethral resection of prostate) to resolve the problem. To minimize the risk of infection, transurethral resection of prostate was performed after laparoscopic transperitoneal access was obtained for repair of the hernia. Bilateral hernia repair with laparoscopy was done by reducing the hernia sac, followed by prosthetic mesh inlay. Mesh type used to close the defect was polypropylene. Preoperative and postoperative levofloxacin single doses were given. The laparoscopic procedures of TEP and TURP were done without any difficulties. The total operating time was 3 h with an estimated blood loss of 100 cc. The last pathological examination before the surgery showed atypical adenomatous hyperplasia with chronic inflammatory cell infiltration, but PSA level was 29 ng/ml. Postoperative follow-up found hematoma in the right inguinal in the third week. An ultrasound examination was performed, and fluid collection was found as shown in Fig. 1. At the three-month postoperative follow-up, the patient had bilateral inguinal abscess and sepsis. Then a CT scan was performed, and the results showed an irregularly shaped mass and prostate (Fig. 2, Fig. 3). Inguinal abscess and post-laparoscopic surgery before are shown in Fig. 4. We conducted evacuation and debridement laparotomy (Fig. 5, Fig. 6). At the next follow-up, no evidence of hernia recurrence after mesh evacuation was found.

## 3 Discussion

Inguinal hernia is one of the most common forms of hernia, found in almost 90% of patients with hernias that occur spontaneously. In the USA, more than 700,000 hernia surgeries are done every year and are the most commonly performed surgical procedures [5]. In recent years, the number of inguinal hernias reported after radical prostatectomy has increased from 7%–21% [[5], [6], [7], [8]]. The study by Stranne et al. [7] showed a comparison of the incidence of inguinal hernias in patients who were operated on vs. non-surgery to be 8.6% vs. 2.4%.

Some laparoscopic techniques for the management of inguinal hernias have been mentioned above, namely TAPP and TEP repair. In both methods, the mesh prosthesis is implanted into the preperitoneal space in the dorsal part of the transversal fascia. Both of these techniques are less invasive versions of the open mesh implantation technique. In TAPP, the surgeon enters the peritoneal cavity and places the mesh through the peritoneal incision above the location of the hernia. However, from the author's perspective, TEP is superior because the peritoneal cavity is not entered, and the mesh is used to close the hernia defect from outside the peritoneum [1].

The incidence of inguinal hernia after prostatectomy is 15–25%. It is estimated that straining during urination coupled with a chronic increase in intra-abdominal pressure is the leading cause of inguinal hernia. The weakening of the inguinal canal wall also increases with age, a risk factor for hernias [4].

One of the advantages of a combination surgery technique between inguinal hernioplasty and TURP is that it saves money, with an average cost of only USD $ 700 in our hospital. The study also suggested that a combination of laparoscopic TURP and hernia repair is easy to do, safe, and effective. The advantage is also that patients only get one anaesthetic for two types of surgery.

Now is the era where the use of synthetic mesh for repair hernias can significantly reduce recurrence. However, the use of synthetic mesh does not mean that the patient cannot experience complications in the mesh. The risk factors of the patient also increase the chances of infection, one of them being elderly. Mesh material and anti-adhesive coating such as polypropylene also appears to affect *Staphylococcus aureus* attachment significantly [9].

Possible causes of infection in the mesh are mesh erosion to the viscera due to fistulisation [10]. Some theories suggest that the formation of biofilms on the mesh is the cause. The biofilm layer protects bacteria from antibiotic exposure and the patient’s immune system, resulting in persistent infections and is increasingly difficult to treat [9,11].

The other possible cause of infection in the mesh are the presence of chronic infection in the prostate. PSA is organ specific (prostate) and it is not specific to prostate cancer. Elevated PSA level in serum is caused by the microarchitecture disruption of prostate gland tissue, which make PSA crossing into surrounding extracellular space. Patients with PSA level >4 ng/ml are considered more at risk of developing prostate cancer. Elevation of PSA level in serum are found in acute, subclinical or chronic prostatitis [13]. This finding is consistent with a study that found local prostate inflammation infiltrates in asymptomatic men with increased PSA level [14]. Biopsy of prostate tissue in this patient shows chronic inflammatory cell infiltration that shows subclinical chronic prostatitis. However, his study is limited to just one case, therefore further research is needed with larger sample with various risk factors.

## 4 Conclusions

In conclusion, the elevation of PSA level can be a warning before doing a combination of TEP and TURP surgery because they can show signs of infection. Another possible cause of infection is the erosion of the mesh to the viscera. Further study with a larger sample size of patients is important to ascertain the possible causes of complication and the relationship between elevated PSA levels and complications of combination TEP and TURP surgery

## Conflict of interest

No potential conflict of interest relevant to this article was reported.

## Sources of funding

The authors declare that this study had no funding resource.

## Ethical approval

The informed consent form was declared that patient data or samples will be used for educational or research purposes. Our institutional review board also do not provide an ethical approval in the form of case report.

## Consent

We have obtained all patient’s consent and had the statement included in the consent section in the manuscript. We also do not include any of the patients name or the institution.

## Author contribution

Adeodatus Yuda Handaya conceived the study. Aditya Rifqi Fauzi and Victor Agastya Pramudya Werdana drafted the manuscript and critically revised the manuscript for important intellectual content. Adeodatus Yuda Handaya, Aditya Rifqi Fauzi, and Victor Agastya Pramudya Werdana facilitated all project-related tasks.

We have obtained all patient’s consent and had the statement included in the consent section in the manuscript. We also do not include any of the patients name or the institution.

## Registration of research studies

There is in literature already published data stating Laparoscopic TEP and TURP from human. This is not the first one.

## Guarantor

Adeodatus Yuda Handaya.

## Provenance and peer review

Not commissioned, externally peer-reviewed.
